# Clinical Analysis of Factors Influencing the Development of Placenta Praevia and Perinatal Outcomes in First-Time Pregnant Patients

**DOI:** 10.3389/fsurg.2022.862655

**Published:** 2022-03-22

**Authors:** Chunhua Zhou, Yang Zhao, Yongmei Li

**Affiliations:** Department of Obstetrics, Renmin Hospital, Hubei University of Medicine, Shiyan, China

**Keywords:** first pregnancy, placenta praevia, risk factors, perinatal period, clinical outcome

## Abstract

**Objective:**

To analyze the risk factors associated with the development of placenta praevia (PP) in first-time pregnant patients and to observe the perinatal clinical outcomes of patients.

**Methods:**

The clinical data of 112 pregnant women with PP (PP group) and 224 pregnant women with normal placental position (general group) who delivered in our hospital from August 2016 to August 2021 were retrospectively analyzed. Baseline demographic data such as age, gestational week, uterine history, assisted reproductive technology use, pregnancy comorbidities, pre-pregnancy body mass index (BMI), smoking, alcohol consumption, placental position, educational level, work were collected from both groups, and logistic regression models were used to analyze the factors influencing the occurrence of PP in patients with first pregnancy. Perinatal outcomes such as implementation of hemostatic treatment (uterine balloon compression, arterial ligation, and B-Lynch suture), maternal postpartum related indicators (amount of postpartum bleeding, incidence of postpartum hemorrhage, blood transfusion rate, blood transfusion volume, and length of hospital stay), and neonatal condition (birth weight, Apgar score at 1 and 5 min after birth) were counted and compared between the two groups.

**Results:**

Histories of endometriosis, use of assisted reproductive technology, and smoking or secondhand smoke inhalation were all high risk factors for PP in patients with first pregnancies, and the proportion of maternal and neonatal adverse outcomes was significantly higher in the PP group than in the general group (*P* < 0.05).

**Conclusion:**

Histories of endometriosis, smoking (secondhand smoke), and use of assisted reproductive technologies are independent risk factors for PP in patients with first pregnancies, which can increase the risk of labor and death of the newborn.

## Preface

Placenta praevia (PP) is one of the most common conditions causing vaginal bleeding during pregnancy and usually occurring after 28 weeks of gestation. It means that the lower edge of the placenta is attached to the lower part of the uterus or reaches (covers) the inner cervical os and the edge of the placenta is lower than the fetal presentation (1). According to the literature, the incidence of PP shows an increasing trend year by year with the increase of cesarean section rate, abortion rate, and uterine operations (2–4). The presence of PP can increase the risk of maternal hemorrhage in late pregnancy, during labor, and in the postpartum period, and in severe cases can lead to hemodynamic instability, reduced oxygen supply, and organ damage causing emergency unscheduled surgery requiring massive blood transfusion; it can also produce serious symptoms such as disseminated intravascular coagulation and multiple organ dysfunction syndrome, which are the main causes of maternal and perinatal death (5, 6).

The exact pathogenesis of PP has not yet been elucidated. Some studies (7–9) suggest that it may be related to damage to the endometrial layer of the uterus due to multiple cesarean sections, multiple abortions, multiple curettage and other uterine operations, and poor blood supply to the placenta during pregnancy; it may also be related to abnormalities in the placenta itself in pregnant patients; it may also be related to the advanced age of the pregnant woman, history of multiple pregnancies and multiple deliveries. In addition, as the frequency of assisted reproductive techniques such as *in vitro* fertilization-embryo transfer increases in infertile patients, the use of ovulation-promoting drugs may also cause the placenta to develop asynchronously with the endometrium, leading to the development of PP. In recent years, the incidence of PP has increased, so it is significant to explore the risk factors associated with the occurrence of PP to reduce the occurrence of adverse maternal and perinatal outcomes (10). However, most of the current studies are on the risk factors associated with the development of PP in patients with a history of prior pregnancy and delivery (11, 12), and there are few reports on the factors influencing the development of PP in first-time pregnant women and the perinatal clinical outcomes. Clinical data show (13) that the incidence of PP in first-time pregnant patients is also increasing year by year, and the etiology is unclear. Based on this, this study analyzed the influencing factors related to the occurrence of PP in first-time pregnant patients and observed the perinatal outcomes such as the implementation of hemostatic treatment, maternal related indicators, and neonatal conditions in both groups. The aim was to identify high-risk factors early so that close monitoring during pregnancy, and active prevention of related complications intraoperatively and postpartum.

## Data and Methods

### Study Population and Grouping

Retrospective analysis of the clinical data of patients who delivered by cesarean section in our hospital from August 2016 to August 2021. Diagnostic criteria (14) for PP: placenta attached to the lower uterine segment reaching or covering the internal cervical os after 28 weeks of gestation and positioned below the previa; also intraoperative diagnosis based on the position of the placenta. **Inclusion criteria:** (1) 28 weeks < pregnancy time < 41 weeks; (2) first pregnancy; (3) singleton with normal fetal position; (4) cesarean section to termination pregnancy; (5) complete case information; (6) meets the diagnostic criteria for PP. **Exclusion criteria:** (1) patients with previous history of miscarriage, cesarean section, or pregnancy; (2) patients with hematologic disorders, malignant tumors, or infectious diseases; (3) previous or existing mental disorders. A total of 336 cases were included in the study, of which 112 pregnant women with PP were counted in the PP group and 224 pregnant women with normal placenta position were counted in the general group.

### Clinical Data Collection

Age, gestational week, uterine history (fibroids, endometriosis), assisted reproductive technology use (yes, no), pregnancy comorbidities (gestational hypertension, gestational diabetes), pre-pregnancy body mass index (BMI), smoking (including secondhand smoke, yes, no), alcohol consumption (yes, no), placental position (anterior, posterior), educational level (Primary and below, Junior–Senior, University and above), work (practitioners, none), and other information. Perinatal outcomes such as implementation of hemostatic treatment (uterine balloon compression, arterial ligation, and B-Lynch suture), maternal postpartum related indicators (amount of postpartum bleeding, incidence of postpartum hemorrhage, blood transfusion rate, blood transfusion volume, and length of hospital stay), and neonatal condition (birth weight, Apgar score at 1 and 5 min after birth) were also counted in both groups.

### Statistical Methods

SPSS 20.0 was used for data processing and analysis, and patients' past medical history, comorbidities, and other count data were described by descriptive statistics such as frequency and percentage (*n*, %), and the χ^2^ test was used for comparison between two groups. Patients' age, BMI and other measurement data were described by statistical indicators such as mean and standard deviation (meam, SD), and *t*-test was used for comparison between two groups. A multifactorial logistic regression model was used to analyse the risk factors associated with the development of PP in first-time pregnant patients. The difference was considered statistically significant at *P* < 0.05.

## Results

### Univariate Analysis of PP in First-Time Pregnant Patients

A comparison of the clinical data of pregnant women in the PP group with those in the general group showed that the proportions of history of uterine fibroids, history of endometriosis, application of assisted reproductive technology, posterior placenta, smoking (secondhand smoke), and alcohol consumption were significantly higher in the PP group compared with the general group (*P* < 0.05). In contrast, there were no statistically significant differences between the two groups in terms of age, gestational week, combined gestational hypertension, combined gestational diabetes, pre-pregnancy BMI, education level, and whether or not they worked (*P* > 0.05), as shown in Table 1.

**Table 1:** Univariate analysis of PP in first-time pregnant patients [mean, SD (*n*, %)].

**Indicators**		**PP group (*n* = 112)**	**General group (*n* = 224)**	***t/χ^2^*** **-value**	***P*** **-value**
Age (years old)		29.46 ± 6.43	30.21 ± 5.22	1.147	0.252
Week of gestation (weeks)		36.26 ± 3.23	35.48 ± 4.51	1.632	0.104
History of uterine fibroids	Yes	7 (6.25)	4 (1.79)	4.699	0.030
	No	105 (93.75)	220 (98.21)		
History of endometriosis	Yes	13 (11.61)	8 (3.57)	8.229	0.004
	No	99 (88.39)	216 (96.43)		
Application of assisted reproductive technology	Yes	10 (8.93)	7 (3.13)	5.236	0.022
	No	102 (91.07)	217 (96.87)		
Combined gestational hypertension	Yes	8 (7.14)	17 (7.59)	0.022	0.883
	No	104 (92.86)	207 (92.41)		
Combined gestational diabetes	Yes	7 (6.25)	15 (6.70)	0.024	0.876
	No	105 (93.75)	209 (93.30)		
Pre-pregnancy BMI (kg/m^2^)		23.37 ± 4.33	24.21 ± 3.58	1.888	0.060
Drinking	Yes	35 (31.25)	43 (19.20)	6.086	0.014
	No	77 (68.75)	181 (80.80)		
Smoking (second-hand smoke)	Yes	63 (56.25)	82 (36.61)	11.744	0.001
	No	49 (43.75)	142 (63.39)		
Placental position	Anterior	45 (40.18)	116 (51.79)	4.031	0.045
	Posterior	67 (59.82)	108 (48.21)		
Educational level	Primary and below	16 (14.29)	29 (12.95)	0.762	0.683
	Junior - Senior	40 (35.71)	91 (40.63)		
	University and above	56 (50.00)	104 (46.43)		
Work	Practitioners	69 (61.61)	130 (58.04)	0.394	0.530
	None	43 (38.39)	94 (41.96)		

### Multifactorial Analysis of PP in First-Time Pregnant Patients

The presence or absence of PP in patients with first pregnancy was used as the dependent variable (Assignment: yes = 0, no = 1), and the indicators that differed in the univariate analysis, such as history of uterine fibroids, history of endometriosis, application of assisted reproductive technology, posterior placenta, smoking (secondhand smoke), and alcohol consumption were used as independent variables (see Table 2 for assignments) into a multifactorial logistic regression model. The results showed that history of endometriosis, application of assisted reproductive technology, and smoking (secondhand smoke) were independent risk factors for the development of PP in patients with first pregnancy (*P* < 0.05, Table 3).

**Table 2:** Assignments.

**Influencing factors**	**Assignment**
History of uterine fibroids	Yes = 0, no = 1
History of endometriosis	Yes = 0, no = 1
Application of assisted reproductive technology	Yes = 0, no = 1
Posterior placenta	Yes = 0, no = 1
Smoking (secondhand smoke)	Yes = 0, no = 1
Alcohol consumption	Yes = 0, no = 1

**Table 3:** Multifactorial analysis of PP in first-time pregnant patients.

**Indicators**	***B***	**SE**	**Walds**	***P***	**OR (95% CI)**
History of uterine fibroids	0.316	0.213	3.058	0.160	1.372 (0.903~2.082)
History of endometriosis	1.324	0.622	32.247	<0.001	3.758 (1.111~12.719)
Application of assisted reproductive technology	1.225	0.438	35.158	<0.001	3.404 (1.443~8.032)
Posterior placenta	0.717	0.634	2.331	0.324	2.048 (0.591~7.097)
Smoking (secondhand smoke)	1.052	0.413	16.138	<0.001	2.866 (1.276~6.440)
Alcohol consumption	0.531	0.463	3.423	0.152	1.701 (0.686~4.214)

### Comparison of the Implementation of Intraoperative Hemostatic Treatment in the Two Groups

**Figure 1:**
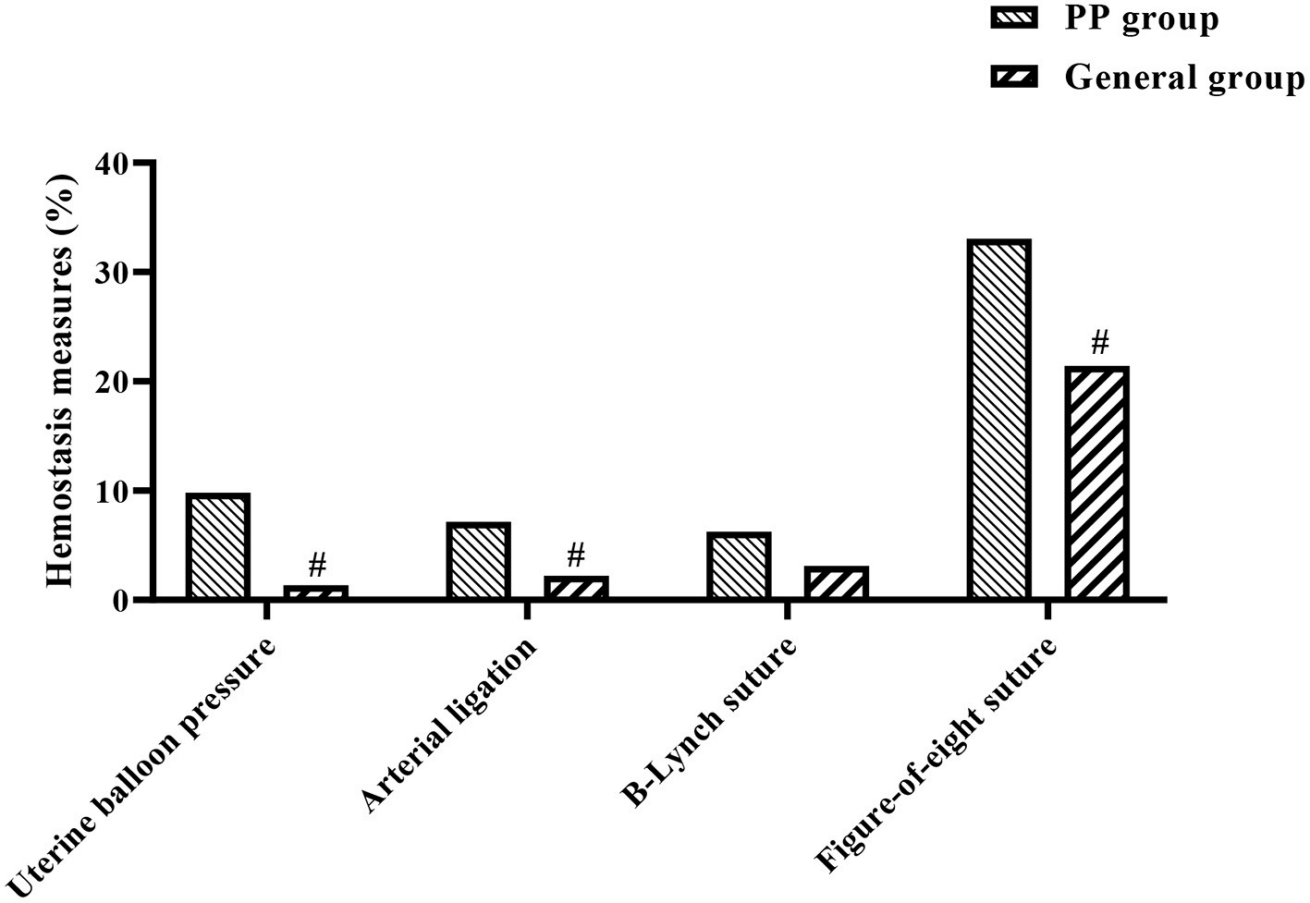
Histogram of the implementation of hemostatic measures in both groups. ^#^indicates comparison between two groups, *P* <0.05.

The probability of patients in the PP group having intraoperative haemostatic treatments such as intrauterine balloon compression, arterial ligation, B-Lynch suture and local figure-of-eight suture was 9.82% (11 cases), 7.14% (8 cases), 6.25% (7 cases), and 33.04% (37 cases), respectively. In the general group, the probabilities were 1.34% (3 cases), 2.23% (5 cases), 3.13% (7 cases), and 21.43% (48 cases), respectively. This shows that the proportion of hemostatic measures such as uterine balloon compression, arterial ligation, and local figure-of-eight sutures performed was significantly higher in the PP group than in the general group (*P* < 0.05; see Figure 1).

### Comparison of Postpartum Conditions Between the Two Groups

**Figure 2:**
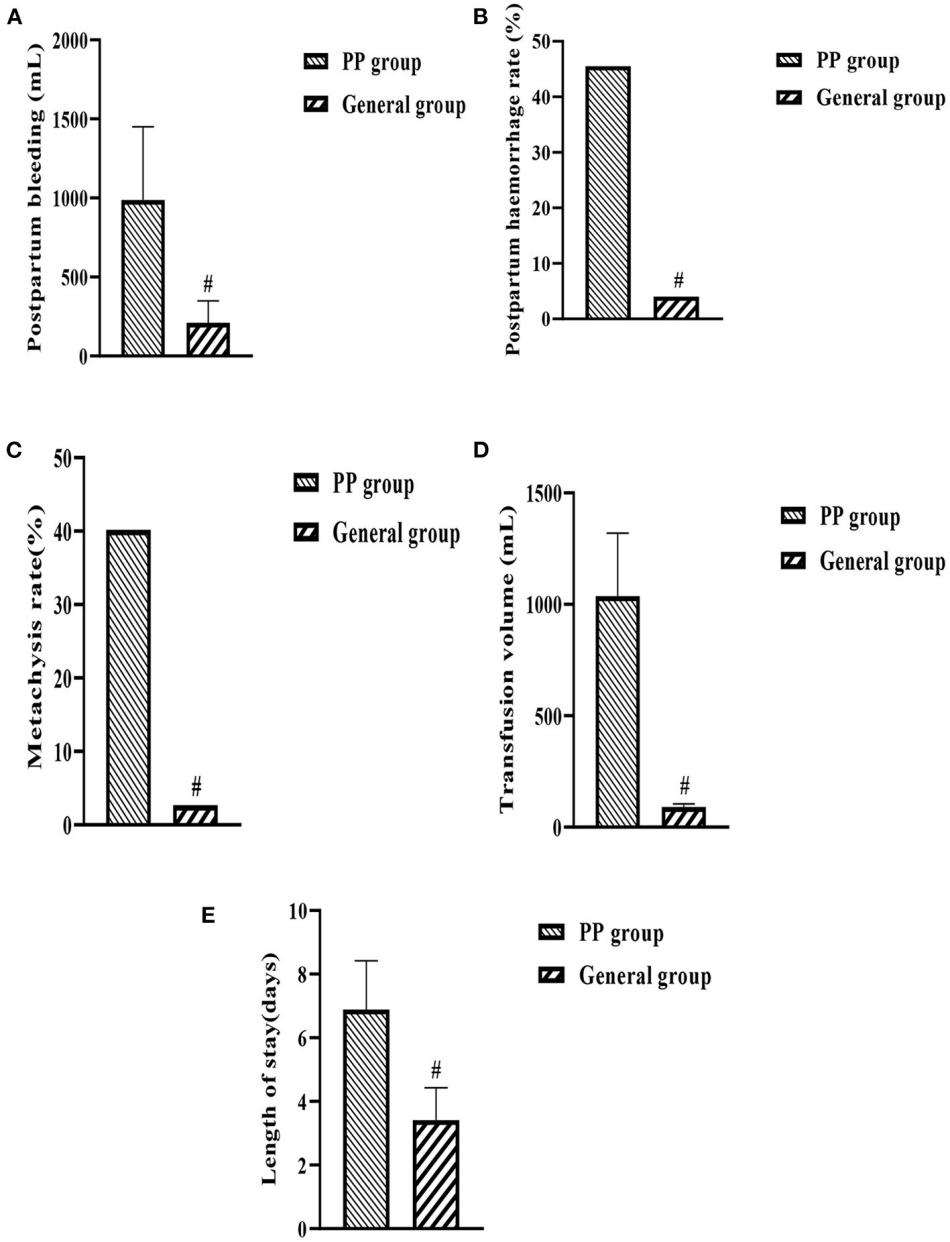
**(A–E)** Histogram of maternal postpartum conditions in both groups. ^#^indicates comparison between two groups, *P* < 0.05.

Postpartum hemorrhage (a), postpartum hemorrhage rate (b), blood transfusion rate (c), blood transfusion volume (d), and hospital stay (e) were significantly at higher levels in the PP group when compared with the general group (*P* < 0.05, Figures 2A–E).

### Comparison of Neonatal Conditions Between the Two Groups

**Figure 3:**
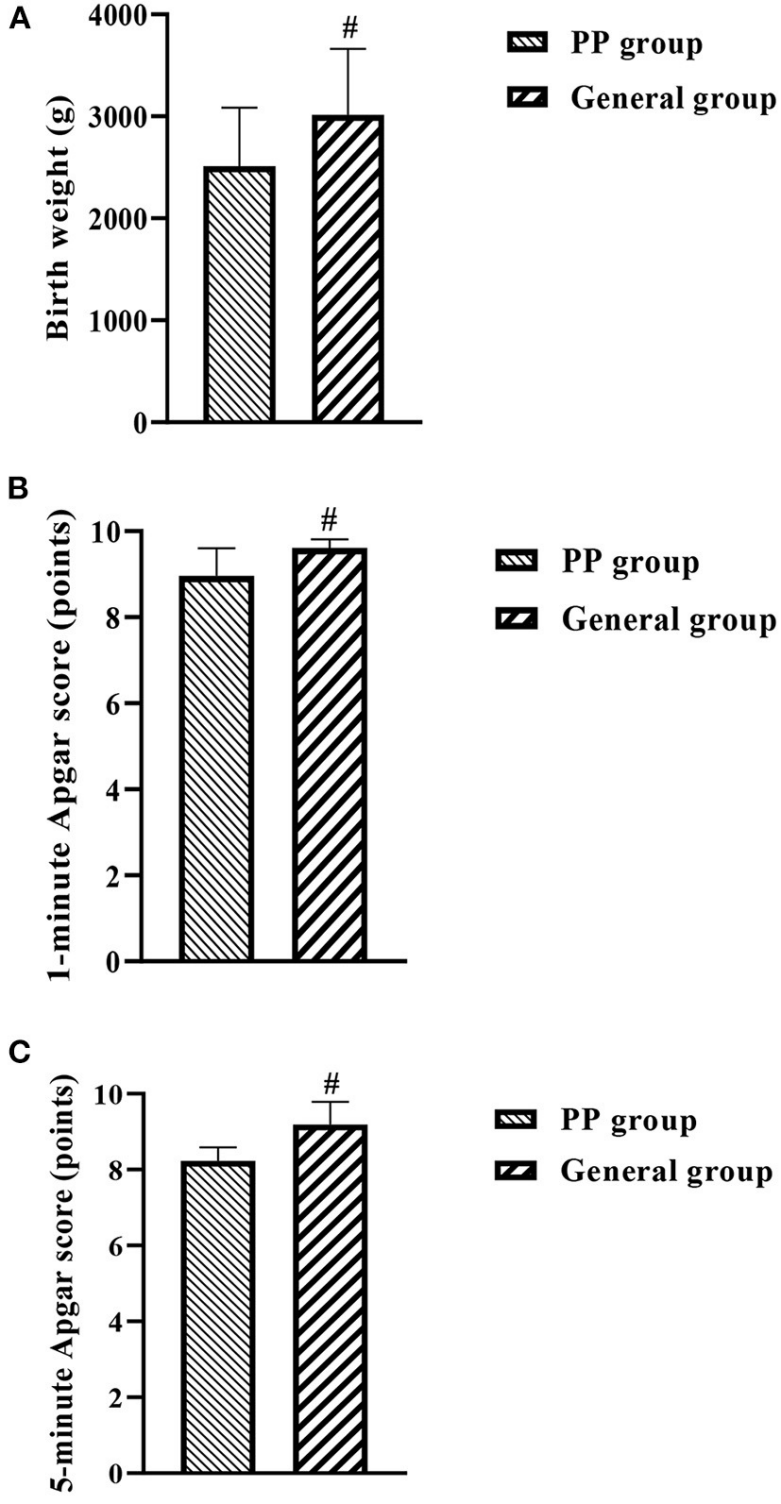
**(A–C)** Histogram of neonatal conditions in both groups. ^#^indicates comparison between two groups, *P* < 0.05.

Neonatal birth weight (a), 1-min Apgar score (b), and 5-min Apgar score (c) were significantly lower in the PP group when compared with the general group (*P* < 0.05, Figures 3A–C).

## Discussion

PP is a common and serious complication of pregnancy and is one of the main causes of maternal hemorrhage in the second trimester, and can also cause maternal postpartum hemorrhage, amniotic fluid embolism, and other adverse events (15, 16). In addition, studies (17, 18) have shown that the incidence of preterm fetal delivery and low birth weight fetuses is significantly higher in women with PP than in those with normal placental position, which shows that the occurrence of PP can also increase the risk of adverse neonatal outcomes, bring serious adverse effects to the family and society. Therefore, it is particularly important to investigate the risk factors associated with the occurrence of PP to reduce the occurrence of adverse maternal and infant outcomes. The current literature (19, 20) mostly examines risk factors for the occurrence of PP in transmaternal women with childbirth history, such as history of miscarriage, history of cesarean delivery, and age. However, reports on the risk factors associated with the development of placenta previa in first-time pregnant patients are rare. Based on the purpose of clarifying the etiology of placenta previa in first-time pregnant women, this study was conducted to observe 336 pregnant women admitted to our hospital as follows.

### History of Endometriosis

This study found that first-time pregnant patients with a history of endometriosis were 3.25 times more likely to develop PP than pregnant women without a history of endometriosis. The results of logistic multivariate analysis indicated that history of endometriosis was an independent risk factor for the development of PP in first-time pregnant patients. Analysis of the reasons for this may be the presence of pelvic adhesions in patients with endometriosis, an abnormally fixed position of the uterus, restricted mobility of the uterus or abnormal contraction of the uterine musculature, which in turn leads to the downward displacement of the placenta to form PP (21). It has been suggested (22) that endometriosis can alter endometrial properties and affect the expression of various factors, especially during the period of placental implantation. It has also been shown (23) that proper progesterone levels play an important role in regulating the endometrium and that endometriosis can cause progesterone resistance, which can lead to the development of PP.

### Application of Assisted Reproductive Technology

The results of this study found that the use of assisted reproductive technology was an independent risk factor for PP in first-time pregnant patients (OR: 3.404, 95% CI: 1.443–8.032). With the widespread application of assisted reproductive technology in clinical practice, the risk of pregnancy related to it has gradually attracted people's attention, and most of the people who use assisted reproductive technology are older, have a history of endometriosis, and have a history of chronic salpingitis (24). In addition to related confounding factors, the application of assisted reproductive technology often requires the use of ovulation-stimulating drugs to change the level of sex hormones in the patients, which may lead to dysregulated or uncontrolled expression of genes related to endometrial turnover, making the asynchrony between endometrial and embryonic development more pronounced and resulting in the formation of PP (25, 26). It has also been suggested (27) that during implantation of the embryo through the mid-uterine cavity using mechanical methods, the transfer tube causes prostaglandin release during passage through the cervical os, leading to uterine contraction, which may result in implantation of the placenta in the lower uterine segment, significantly increasing the probability of PP.

### Smoking or Inhalation of Second-Hand Smoke

Whether smoking or inhalation of secondhand smoke is a risk factor for PP has been controversial in previous national and international studies. A study by Hung et al. (28) confirmed that the risk of PP due to smoking or passive smoking is 2.2 times higher than that of non-smoking or passive smoking. The results of this study showed that smoking or secondhand smoke inhalation was an independent risk factor for PP in first-time pregnant patients (OR: 2.866, 95% CI: 1.276–6.440). Analyzing the reasons for this, exposure to nicotine and carbon monoxide when pregnant women smoke or inhale secondhand smoke can cause chronic placental hypoxia, and placental hypoxia can lead to necrosis in the decidua of the uterus and microthrombus formation in the placenta, presumably extending and expanding to cover the endocervix in order to obtain more nutrients leading to PP (29, 30).

In addition, the study also looked at maternal and neonatal conditions in both groups and showed that intraoperative haemostatic measures such as intrauterine balloon compression, arterial ligation and local figure-of-eight sutures were used significantly more frequently in the PP group than in the general group of pregnant women. Postpartum hemorrhage, postpartum hemorrhage, blood transfusion rate, transfusion volume, and length of stay were significantly higher in the PP group compared to the general group, while the PP group had lower birth weight, 1 and 5 min Apgar scores. This suggests that the occurrence of PP has a more detrimental effect on both the mother and the neonate, increasing the risk of labor, as well as causing respiratory distress in the neonate and increasing the risk of neonatal death.

In summary, histories of endometriosis, use of assisted reproductive technology, and smoking or secondhand smoke inhalation are all high-risk factors for PP in first-time pregnant patients. Therefore, we should strengthen the promotion of reproductive health information, avoid smoking or secondhand smoke inhalation in pregnant women, and pay close attention to patients with these risk factors in clinical practice, and take appropriate measures to improve the outcome of patients and newborns.

## Data Availability Statement

The original contributions presented in the study are included in the article/supplementary material, further inquiries can be directed to the corresponding author/s.

## Ethics Statement

The studies involving human participants were reviewed and approved by the Local Medical Ethics Committee. The patients/participants provided their written informed consent to participate in this study.

## Author Contributions

All authors contributed to this experiment, including experimental design, case collection, data analysis, and paper writing. All authors read and agreed to the final version.

## Conflict of Interest

The authors declare that the research was conducted in the absence of any commercial or financial relationships that could be construed as a potential conflict of interest.

## Publisher's Note

All claims expressed in this article are solely those of the authors and do not necessarily represent those of their affiliated organizations, or those of the publisher, the editors and the reviewers. Any product that may be evaluated in this article, or claim that may be made by its manufacturer, is not guaranteed or endorsed by the publisher.
